# SAR, Molecular Docking and Molecular Dynamic Simulation of Natural Inhibitors against SARS-CoV-2 Mpro Spike Protein

**DOI:** 10.3390/molecules29051144

**Published:** 2024-03-04

**Authors:** Aqsa Salamat, Naveen Kosar, Ayesha Mohyuddin, Muhammad Imran, Muhammad Nauman Zahid, Tariq Mahmood

**Affiliations:** 1 Department of Chemistry, University of Management and Technology (UMT), C-II, Johar Town, Lahore 54770, Pakistan; S2017263003@umt.edu.pk (A.S.); naveen.kosar@umt.edu.pk (N.K.); ayesha.mohyuddin@umt.edu.pk (A.M.); 2 Department of Chemistry, Faculty of Science, King Khalid University, P.O. Box 9004, Abha 61413, Saudi Arabia; miahmad@kku.edu.sa; 3 Department of Biology, College of Science, University of Bahrain, Sakhir P.O. Box 32038, Bahrain; 4 Department of Chemistry, COMSATS University Islamabad, Abbottabad Campus, Abbottabad 22060, Pakistan; 5 Department of Chemistry, College of Science, University of Bahrain, Sakhir P.O. Box 32038, Bahrain

**Keywords:** COVID-19, density functional theory (DFT), molecular docking (MD), molecular dynamics simulation, structure–activity relationship (SAR), pharmacokinetics

## Abstract

The SARS-CoV-2 virus and its mutations have affected human health globally and created significant danger for the health of people all around the world. To cure this virus, the human Angiotensin Converting Enzyme-2 (ACE2) receptor, the SARS-CoV-2 main protease (Mpro), and spike proteins were found to be likely candidates for the synthesis of novel therapeutic drug. In the past, proteins were capable of engaging in interaction with a wide variety of ligands, including both manmade and plant-derived small molecules. *Pyrus communis* L., *Ginko bibola*, *Carica papaya*, *Syrian rue*, and *Pimenta dioica* were some of the plant species that were studied for their tendency to interact with SARS-CoV-2 main protease (Mpro) in this research project (6LU7). This scenario investigates the geometry, electronic, and thermodynamic properties computationally. Assessing the intermolecular forces of phytochemicals with the targets of the SARS-CoV-2 Mpro spike protein (SP) resulted in the recognition of a compound, kaempferol, as the most potent binding ligand, −7.7 kcal mol^−1^. Kaempferol interacted with ASP-187, CYS-145, SER-144, LEU 141, MET-165, and GLU-166 residues. Through additional molecular dynamic simulations, the stability of ligand–protein interactions was assessed for 100 ns. GLU-166 remained intact with 33% contact strength with phenolic OH group. We noted a change in torsional conformation, and the molecular dynamics simulation showed a potential variation in the range from 3.36 to 7.44 against a 45–50-degree angle rotation. SAR, pharmacokinetics, and drug-likeness characteristic investigations showed that kaempferol may be the suitable candidate to serve as a model for designing and developing new anti-COVID-19 medicines.

## 1. Introduction

Due to the deadly contagiousness virus of SARS-CoV-2, the COVID-19 outbreak has posed a significant threat to global health [1]. Unfortunately, the pandemic has had serious negative impacts on every aspect of society throughout the world [2]. Dry cough, fever, tiredness, dyspnea, and shortness of breath are the main symptoms of COVID-19 [3]. SARS-CoV-2 primarily disseminates through the air, respiratory aerosols, and droplets generated by infected individuals during actions such as coughing, sneezing, talking, and breathing. Transmission can occur through direct or indirect contact, including instances such as the fecal–oral route [4]. The virus affects multiple organs by targeting the lining of the respiratory tracts [5], kidney, liver, gut, and cardiovascular organs, and as well as the nervous system. In immune-compromised individuals who have diabetes [6], obesity, coronary heart diseases, and hypertension, severe complications are frequently seen [7]. A diversity of SARS-CoV-2 variations, including double- and triple-mutant strains in some regions, have been documented worldwide [8]. These variations are quite infectious, as their infectivity and transmission rate is higher [9]. There is still some uncertainty over the ability of the existing vaccines to effectively prevent them, as they can lead to re-infection [10]. Because COVID-19 can rapidly exhibit modification in its spike protein receptor binding region, the signs of virus and clinical manifestations are complicated throughout the world [11].

Natural compounds are the most promising choices in this area, as they have low cytotoxicity and high bioavailability [12]. Since prehistoric days, humans have relied on natural compounds, particularly phytochemicals, to treat various ailments and disorders. Flavonoids are secondary metabolites produced by plants; they serve vital functions in plant metabolism and have several potential biological advantages, including antioxidant, anti-inflammatory, anticancer, antibacterial, antifungal, and antiviral activity [12]. The potential antiviral activities of several flavonoids, including flavones and flavonols, have been comprehensively studied. It is claimed that medicinal plants represent the primary source of healthcare for approximately 85 percent of the population, and more than 40 percent of synthetic medications on the pharmaceutical market are extracted from plants and microbial-based natural products [13].

**Figure 1:**
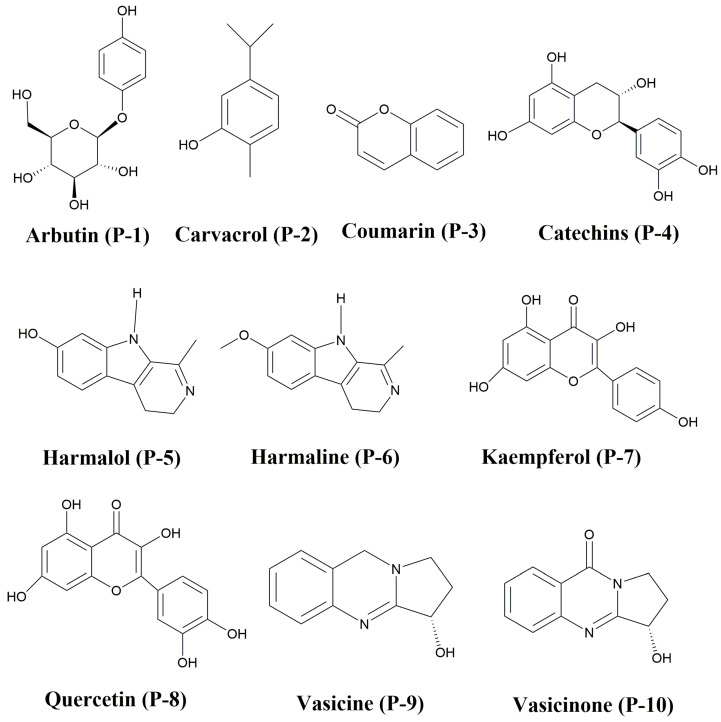
Chemical structures of phytochemicals P-1 to P-10.

This report discusses the importance of natural compounds against COVID-19. Computer-aided approaches are adopted in search of some important phytochemicals against COVID-19 [14]. Approaching the binding sites of SARS-COV-2 main protease and spike proteins with different phytochemicals was the principal focus of this research. The selected phytochemicals include arbutin (P-1) from *Pyrus communis* L. [15]; catechin (P-4) and carvacrol (P-2) from *Ginko bibola* [16]; coumarin (P-3) from *Pimenta dioica* [17]; quercetin (P-8) and kaempferol (P-7) from *Carica papaya* [18]; and vesicine (P-9), vesicinone (P-10), harmaline (P-6), and harmalol (P-5) from *Syrian rue* [19] (Figure 1). Ten phytochemicals from three distinct families showing different binding affinities were selected for the current study.

## 2. Results and Discussion

### 2.1. Ligand Modeling Studies (LMSs)

Calculations based on the density functional theory (DFT) and Gaussian interphase version 9.0 were utilized to obtain the energy minima structures of the investigated phytochemicals [20]. Figure 1 represents the chemical structures of all ten selected phytochemicals.

**Figure 2:**
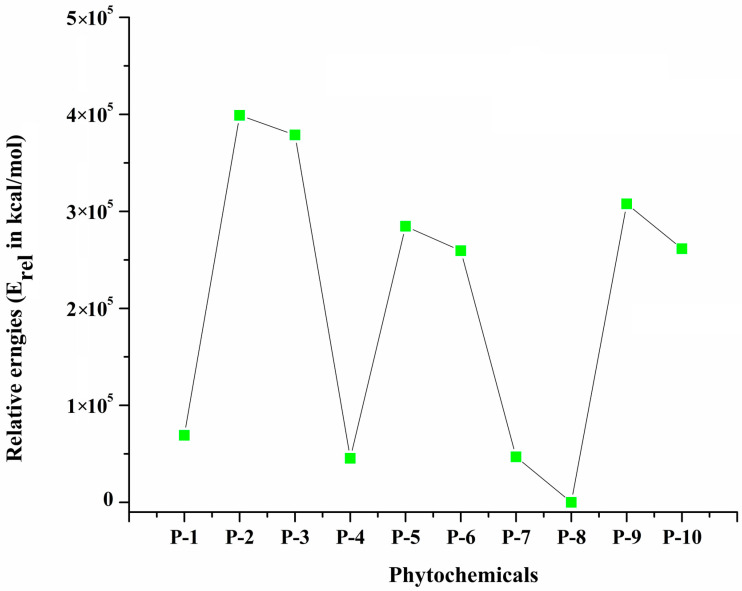
Relative energies (E_rel_ in kcal/mol) of all selected phytochemicals (P-1 to P-10).

The relative energy (E_rel_) of the optimized geometries of all selected phytochemicals is used to describe comparative stability. Figure 2 shows the relative stability of each phytochemical with respect to E_rel_. P-8 is the most stable phytochemical, whereas (P-2) is least stable phytochemical because of its relative energy (4.55 × 10^4^ kcal/mol), and hence, it is more reactive. Other phytochemicals show the average stability. The dipole moment values obtained for P-1, P-4, P-5, P-7, P-8, and P-9, were higher compared to those of P-2, P-3, and P-10. P-7 has a greater dipole moment (5.38 Debye), while P-10 shows the lowest value (0.81 Debye) of dipole moment. All phytochemicals exhibited intermolecular interactions with 6LU7 at different active sites and at variable binding strengths.

### 2.2. Frontiers Molecular Orbitals (FMOs) and Reactivity Descriptors of Selected Phytochemicals

A FMO analysis was performed for selected compounds to determine the reactivity [21], and the results are compiled in Table 1. HOMO and LUMO variations explain the fundamental charge transfer (CT) interaction. Here, HOMO-LUMO energy difference refers to the reactivity and stability of selected phytochemicals. It was discovered that P-8 has the smallest HOMO-LUMO energy gap (3.48 eV) among all phytochemicals. The obtained HOMO-LUMO energy gap value indicates that P-8 is the most reactive, while P-1 has the highest HOMO-LUMO energy gap (5.60 eV), so it is the most stable among all phytochemicals.

A variety of chemical reactivity descriptors, including the electronic chemical potential, electronegativity, softness, hardness, and electrophilicity index, were calculated to provide conclusive evidence about the reactivity of considered phytochemicals. The reactivity of phytochemical increases as its chemical hardness value decreases, whereas its stability increases as its chemical hardness value increases [22]. The results indicate that P-8 has the lowest chemical hardness (*η*) value (−1.74 eV), so it is most reactive. Meanwhile, P-1 has the highest chemical hardness (−2.80 eV), which depicts the highest stability of the respective compounds. The electrophilicity value (ω) is another crucial factor, with positive measuring the transfer of electrons from the surrounding environment into the system. We found that P-1 was the most stable molecule due to its reduced electrophilicity (2.02 eV) while the P-8 has a maximum electrophilicity value (4.24 eV) that indicates its highest reactivity. The electrochemical chemical potential (*µ*) of all phytochemicals is in the range from −2.78 to −4.21 eV. P-3 has highest *µ* value of −4.21 eV; on the other side, P-4 has the lowest *µ* value of −2.78 eV among all phytochemicals. The nucleophilicity index (N) is also an important parameter for understanding the reactivity of compounds. All the considered compounds have nucleophilicity values in the range from −3.48 to −5.60 eV. The compound P-8 had the lowest nucleophilicity value (−3.48 eV), which represents the soft nucleophilic nature of respective compound, whereas compound P-1 has the highest N value (−5.60 eV), which indicates the strong nucleophilic nature of this compound.

### 2.3. Structure and Activity Relationship (SAR) Analysis

The structure and activity relationship (SAR) was used to anticipate the activity with respect to the structure of the selected phytochemicals by using HyperChem Professional 8.0.3 software [19]. The Fletcher–Reeves conjugate gradient algorithm method was employed for energy minimization; then, all compounds were optimized by utilizing the semi-empirical PM3 method. Different parameters, such as the surface area (A^2^), volume (A^3^), hydration energy (kcal/mole), refractivity, and polarizability (Å^3^), of selected chemicals were calculated, showing considerable variances in their physical and chemical properties (Table 2).

The surface area and volume provide insights into the molecular size and shape of the molecule, influencing their interactions with biological targets. P-9 has the smallest volume, indicating a relatively compact molecular structure. In contrast, P-3 exhibits a larger volume and surface area, suggesting increased binding affinities and efficacy due to its ability to interact with multiple binding sites with target molecules. Hydration energy reflects the extent of interaction between the compound and water molecules, which is essential for predicting their solubility and bioavailability. P-3, P-4, and P-5 exhibit profoundly higher negative hydration energy values, indicating significant interactions with water molecules. In contrast, P-7 has a considerably less negative hydration energy value, suggesting its weaker interactions.

The log *p*-value indicates the hydrophobicity or lipophilicity of compounds, influencing their ability to cross biological membranes and to penetrate the targeted cells. Based on the log *p*-value, P-3 emerged as being hydrophobic in nature, but P-1 and P-9 were found to be hydrophilic. Furthermore, P-3 has the greatest refractivity and polarizability, significantly impacting light bending, high electron density and potential for strong molecular interactions. P-9, on the other hand, has the lowest reflectivity and polarizability, suggesting that it has less influence on these characteristics. Compounds with higher refractivity and polarizability exhibit more vital interaction with target molecules, potentially leading to increased potency and efficacy. These properties provide helpful information for drug development and molecular modeling applications.

### 2.4. Molecular Docking Study

Molecular docking was utilized to estimate the interactions between protein and the considered phytochemicals [23]. The docking analysis was executed by using the 3D crystal structure of SARS-CoV-2 Mpro (6LU7) as a biological target. We examined ten different phytochemicals derived from various natural sources. All phytochemicals interacted with the main protease (Mpro) of SARS-COVID-19 by docking in the cavity of the binding pocket, which contains common amino acid residues, such as ARG-131, LYS-137, THR-199, TYR-239, and LEU-288. The binding affinity values of the three most stable orientations for each phytochemical with the target are provided in Table 3. In the discussion, we focus only on the most stable orientation due to its energetic feasibility. The docking (binding free energy) scores of the first most stable orientation ranges from −4.7 to −7.7 kcal/mol for all phytochemicals. Here, P-7 has the highest binding energy (−7.7 kcal/mol), and P-2 has the lowest binding energy (−4.7 kcal/mol).

**Figure 3:**
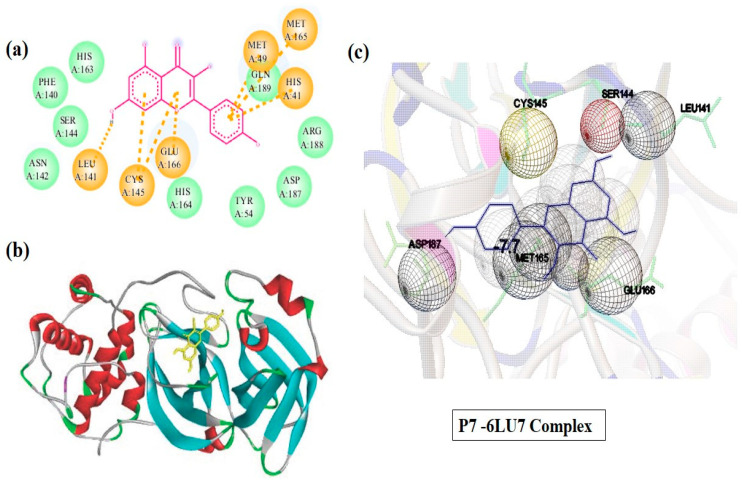
Two-dimensional (**a**), three-dimensional (**b**), and residue interactions analysis (**c**) based on structures of P-7-6LU7 complex.

The most stable orientation was selected for molecular docking. Moreover, the molecular docking outcome revealed that all the chosen phytochemicals could effectively bind with the Mpro of SARS-CoV-2. The most crucial factor for the activity of molecules is the binding energy between the ligand and the target protein [24]. According to the results, compound P-7 (kaempferol) had the highest binding energy of −7.7 kcal/mol, which could be attributed to its aromatic ring structure and hydroxyl groups that form hydrogen bonding with amino acid residues (LEU-141, CYS-145, GLU-166, HIS-41, MET-49, GLN-189, and MET 165) of Mpro. Similarly, compound P-8 (quercetin) also has reasonable binding energy, i.e., −7.5 kcal/mol, owing to its hydrogen bonding with the amino acid residues of Mpro. On the other hand, P-2 (Carvacrol) showed the lowest binding energy of −4.7 kcal/mol, which could be attributed to its small size and lack of specific functional groups that can form stable interactions with the amino acid residues in the binding sites. Detailed interactions of the P-7-6LU7 complex are illustrated in Figure 3. The structures of the remaining complexes, other than P-7, are given in Supplementary Materials Figure S1.

### 2.5. Pharmacokinetics and Pharmacodynamics Predictions of Selected Phytochemicals

Pharmacokinetics and pharmacodynamics can be utilized to design a prospective small drug that could be used for a particular target. Using the Lipinski Rule of Five, these ten compounds’ canonical SMILES were obtained from PubChems database and further assessed for drug-likeness properties. According to this criterion, for a chemical to exhibit drug-like characteristics, it must have a molecular weight of <500 Da, less than five H-bond donors (HBDs), less than ten H-bond acceptors (HBAs), and a lipophilicity of log *p* < 5 [25]. The compound of interest can be a potential candidate for new drug development if it meets more than two of the abovementioned requirements. All phytochemicals obeyed Lipinski’s Rule of Five (Ro5) [26]. Table 4 presents the *in-silico* analysis of the pharmacokinetics and pharmacodynamics properties of phytochemicals (P-1 to P-10). The molecular weight (MW) of all phytochemicals was less than 500 Da, which is the maximum limit as per Lipinski’s Rule of Five (Ro5) for exhibiting the drug-like properties. All compounds had less than five hydrogen bond donors (HBDs) and less than seven hydrogen bond acceptors (HBAs), fulfilling the criteria for drug-like characteristics. The number of rotatable bonds (RBs) was less than three in all compounds except for P-1, indicating good flexibility of the ligands [27]. The Lipinski filter was further assessed for the solubility of these compounds. Solubility is the ability of a compound to dissolve in physiological fluids, as it affects the absorption, distribution, and bioavailability within the body. The estimated solubility (ESOL Log S) and estimated aqueous solubility (ESOL) were investigated to predict the aqueous solubility of the considered phytochemicals. P-1 has the highest ESOL Log S values (−0.71), indicating better solubility in water, while values for P-7 and P-8 have lowest solubility value of −3–31 and −3.16, respectively. The Log S values for all other phytochemicals were negative, indicating their hydrophobic nature. These outcomes are similar to those of the work of Jin et al. on kaempferol’s medicinal activity [28]. The bioavailability score for all phytochemicals (P-1 to P-10) is 0.55, indicating their efficient absorption and distribution within the body, as reported by Arts et al. [29]. The gastrointestinal (GI) absorption value of all phytochemicals was high, indicating that they could be easily absorbed in the gastrointestinal tract. The synthetic accessibility (SA_score_) of phytochemicals was in the range of 1–4.18, indicating that they could be synthesized easily. Finally, the compounds were analyzed for their ability to cross the blood–brain barrier (BBB). P-2, P-3, P-5, and P-6 showed lower BBB permeability than others which implies that these compounds may not be able to penetrate into BBB effectively. Overall, the results of the in silico analysis suggest that all phytochemicals have good drug-likeness properties and are potential candidates for further development as drugs.

### 2.6. Toxicity Analysis by ProTox

Predictions of toxicity and suitable biological properties play a crucial role in the drug-designing phase. The selected phytochemicals from SWISS ADME were investigated further in ProTox 1.6.4. This tool was used to evaluate the physicokinetic properties that explain the distribution of various compounds within the body. ProTox was used to evaluate the toxicity and biological properties of the ten investigated phytochemicals (P-1–P-10) from ADMET. The results revealed that all phytochemicals were inactive, except for P-4 and P-9. P-4 was inactive against hepatotoxicity and immunotoxicity, while P-9 was inactive against mutagenicity [30].

### 2.7. MD Simulation

Molecular dynamics (MD) simulations were conducted for the optimal complex P-7-6LU7. The ligand–protein complexes underwent a simulation of 100 ns to assess the root-mean-square deviation (RMSD) and root-mean-square fluctuation (RMSF). The RMSD simulation plot showed that protein fluctuated a little, i.e., from 2.35 Å to 2.69 Å, for only 6ns in a frame/time (2283/45.6–2698/53.95). In the last frame, there was not a significant fluctuation. On the other side, ligands remained equilibrated throughout the simulation; however, a 14.0 Å displacement was observed in frame/time (2508/50.15–3072/61.43). A ligand returns to equilibration within 8ns till the end of simulation. The ligand–protein complex remained under the 1–3 displacement limit. CYS-44 and GLY-195 showed a maximum fluctuation of 2.34–2.23 Å, respectively, whereas TYR-54, ARG-60, LYS-61, ASN-119 THR169, ASN-214, ARG-222, GLN-244, and SER-284 exhibited a little conformational change in the range of 1.18–1.25 Å. Heavy atom 2 showed maximum displacement of 15.3 Å when fitted on protein, while atoms 6, 8 11, and 19 reflected a displacement in the range of 14.8–14.9 Å. Meanwhile, the rest of the atoms did not exhibit any conformational change for protein–ligand displacement, which was 14.2 Å. Throughout the simulation, the root-mean-square fluctuation (RMSF) consistently maintained a value below 2.5 Å. For small globular proteins, deviations within 1–3 Å are permissible. This complex can be further analyzed for protein–ligand contact.

**Figure 4:**
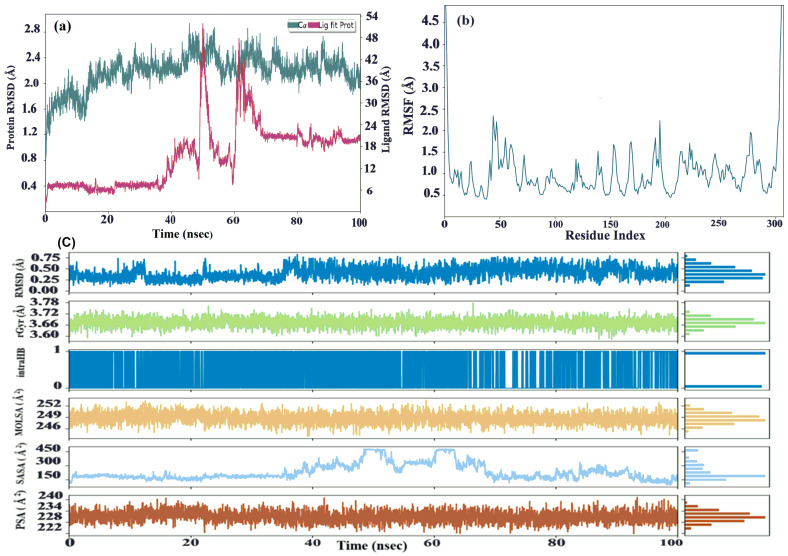
Root-mean-square deviation (RMSD) (**a**). Root-mean-square fluctuation (**b**). Molecular Surface Area (MolSA), Solvent-Accessible Surface Area (SASA), Radius of Gyration (rGy), and Polar Surface Area (PSA) (**c**) of the (P-7-6LU7) complex.

Calculations of the protein–ligand root-mean-square deviation (RMSD), Molecular Surface Area (MolSA), Radius of Gyration (rGy), Polar Surface Area (PSA), and Solvent-Accessible Surface Area (SASA) were used to evaluate the ligand’s properties (see Figure 4). For P-7-6LU7, the RMSD of the ligands initially varied to a maximum of 8.1 ns before reaching equilibrium. The RMSD values of the ligands varied between 0.28 and 0.54 Å, with equilibrium occurring at 0.28 Å at 39.1 ns (P-7-6LU7). The ligands’ rGyr fluctuated marginally before achieving equilibrium. The ligands were in equilibrium at 3.69 Å at 2.08 ns (P-7-6LU7), with rGyr values ranging between 3.66 and 3.69 Å. Between atoms 21 and 2(O–H----O=C), there is only one intra hydrogen bond of 51% strength (intraHB). MolSA values for the ligands range from 246.4 to 252.06 Å^2^, with equilibrium at 249.4 Å^2^ (P-7-6LU7).

The Solvent-Accessible Surface Area (SASA) refers to the area of the surface of the ligand which is accessible to solvent molecules and indicates how much of the ligand is exposed to the surrounding environment. The SASA values of the ligand varied between 230.77 and 371.4 A^2^, indicating fluctuations in the accessibility of the ligand within the protein’s active site. Initially, the SASA values of the ligands fluctuated slightly before reaching equilibrium. This may be due to the ligand initially reorienting itself within the active site. Eventually, the ligand settled and attained a stable conformation, with an SASA value of 181.2 Å^2^ at the equilibrium (P-7-6LU7) complex.

During the simulation, the ligand’s ability to interact with polar solvent molecules was measured by its solvent Polar Surface Area (PSA). The PSA remained constant, indicating that the ligand had consistent and stable interactions with the solvent molecules. The equilibrium value of 234 Å^2^ shows that there was a balanced distribution of polar contacts, which contributed to the stability of the protein–ligand complex. The ligands’ PSA values ranged between 230.6 and 233.42 Å^2^, which means that the properties of the ligand initially changed slightly but attained equilibrium and remained constant throughout the simulation. This demonstrates that the ligand was stable in the protein’s active region.

**Figure 5:**
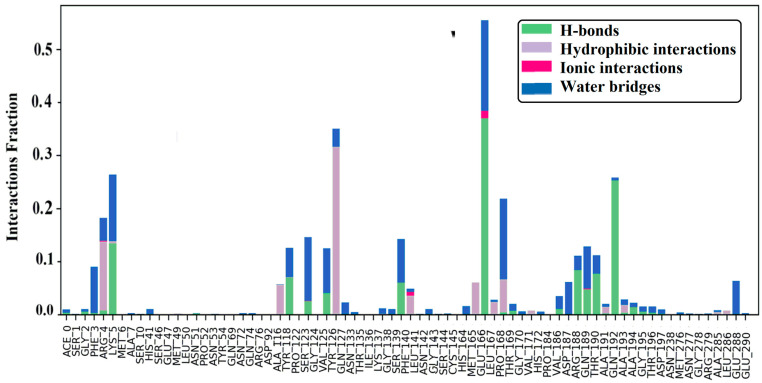
Protein–ligand contact and interaction plots.

The protein–ligand (P-L) contact histograms of the stable complex and interaction residues are illustrated in (Figure 5). There are four types of P-L interactions: hydrophobic, ionic, hydrogen bonds, and water bridges. As hydrogen bonds are one of the important interactions for ligand binding, they must be considered when designing drugs. PHE-3, ARG-5, LYS-5, TYR-118, SER-123, VAL-125, SER-139 VAL-186, ASP-187, ARG-188, GLN-189, THR-190, GLN-192, and GLU-288 formed hydrogen bonding with ligands in the fraction range of 0.235–0.008. GLN-192 has a polar amide side chain, so it can directly form hydrogen bonds, behaving as both donor and acceptor. GLU-188 has the strongest hydrogen bond interaction with ligands among all amino acids (see Figure 5). The amino acid residues, including ARG-4, ALA-116 TYR-126, PHE-140, MET-165, LEU-167, PRO-168, ALA-191, and ALA-193, show hydrophobic interactions. ARG-4, SER-123, PHE-140, and GLU-166 formed ionic bonding, and GLU-166 demonstrated strong ionic bonding. Through water-bridging, ACE-0, GLY-2, PHE-3, ARG-4, GLU-288, TYR-118, SER-123, VAL-125, TYR-126, GLN-127, ASN-133, LYS-137, GLY-138, ASN-142, GLU-166, PRO-168, THR-169, GLY-170, VAL-186, ARG-188, GLN-189, THR-190, ALA-191, ALA-193, ALA-194, GLY-195, THR-196, ASP-197, ALA-285, GLU-288, and GLU-290 were bonded. The highest water-bridging was seen between GLU-166 and a ligand. Similarly, other amino acids, such as positively charged ARG-188, LYS-5, hydrophilic SER-139, and SER-139 and negatively charged ASP-187 and GLU-288, facilitate Van der Waals interactions with ligands in the protein–ligand complex. These results indicate that amino acid residues interacted with ligands in every conceivable orientation, with considered interactions which give overall stability to the P7-6LU7 complex.

**Figure 6:**
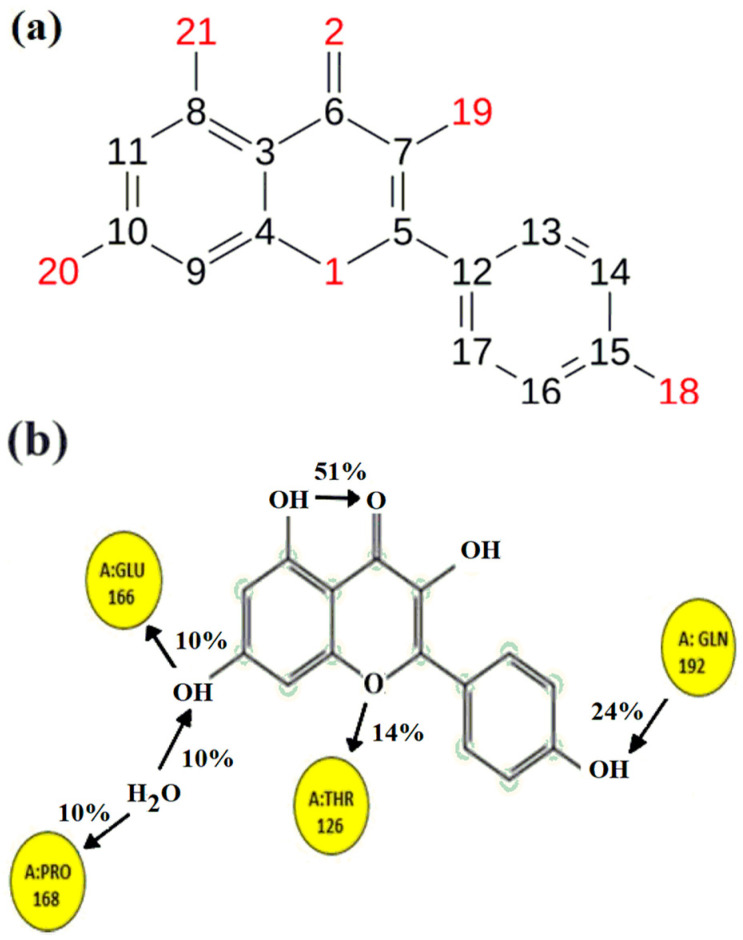
(**a**) Represent the numbering scheme of the Kaempferol P-7 and (**b**) represent interactions of the functional groups within the P-7 ligand with the specific amino acids of the protein.

The weakest contact strength (10%), as demonstrated in Figure 6, belonged to negatively charged GLU-166, which accepted a proton from atom 20 and a proton from a hydrogen-bonded water molecule to PRO-168. TYR-126 interacted with the central heterocyclic through π electrons, while the terminal atom 18 accepted the proton from GLN-192 with a 24% contact strength. Between atoms 21 and 2, an intra-hydrogen bond was observed. Interactions decrease at a greater contact strength (90%); only intra-hydrogen bonds remain intact.

**Figure 7:**
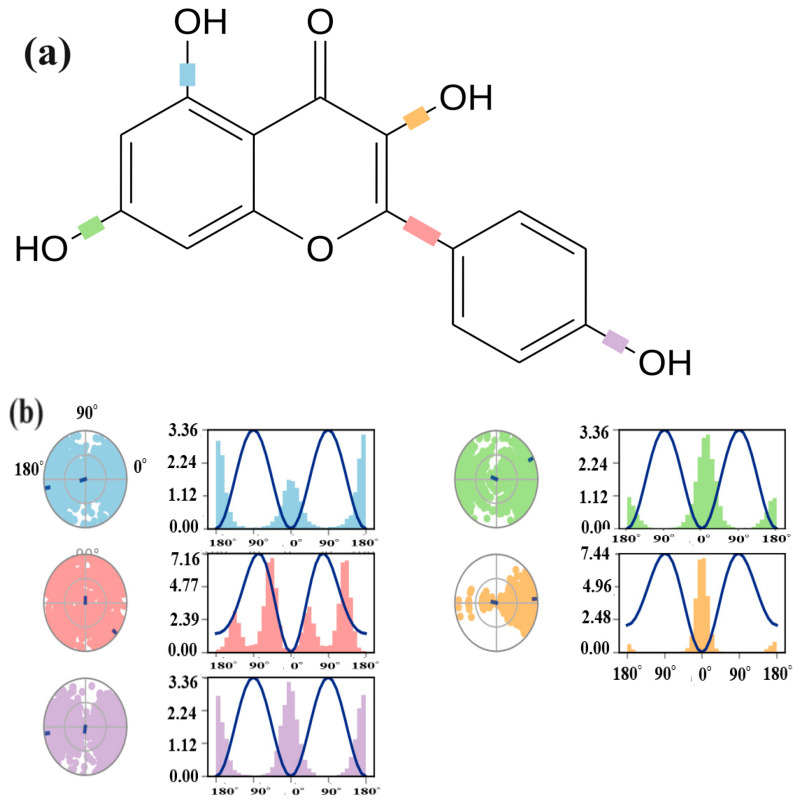
(**a**) Shows the 2D schematic of a ligand with different color-coded rotatable bonds. Each rotatable bond torsion is accompanied by a dial plot and bar plots of the same color in (**b**). Where dial plots describe the conformation of the torsion throughout the course of the simulation. The bar plots summarize the data on the dial plots, by showing the probability density of the torsion. The values of the potential of the rotatable bond are on the left Y-axis of the chart, and are expressed in kcal/mol.

The torsional degree of freedom refers to the rotation around the single bond within the molecule. It allows the ligand to adopt an optimal orientation for binding to the protein. The values for the torsional degree were calculated for the P-7-6LU7 complex. During the trajectory simulation, variations in specific bonds within the ligand were observed. As shown in (a) part of Figure 7, four C–O and one C–C bond exhibited variations in the torsion conformation within the ligand during trajectory simulation. Bonds between atoms 8 and 21 exhibited three distinct conformations, while bonds between atoms 5 and 12 exhibited four distinct conformations, two with major and two with minor rotational angles. This result indicates the presence of distinct conformation adopted by kaempferol within the binding pocket of 6LU7. The potential differences between 8 and 21; 10 and 20; and 15 and 18 were 3.36 units, while the potential difference between 5 and 12 was 7.16. Atoms 7 and 9 exhibited one main conformation up to 40 degrees of rotation and 7.44 potential units, suggesting specific molecular arrangements that contribute significantly to overall binding affinity, whereas the other two conformations were minimal and possessed a potential change of only 0.1 units, indicating less stable binding modes (see Figure 7).

## 3. Materials and Methods

### 3.1. Density Functional Theory (DFT) Methods

First, the Gauss View 5.0 program [31] was used for the visualization of results, and Gaussian 09 [32] was implemented for the density functional theory (DFT) calculations. B3LYP, a hybrid functional of DFT, was used, along with a 6-31G (d, p) basis set, for the optimization of selected phytochemicals. This method (B3LYP/6-31G(d, p)) has already reported for the optimization of some other important natural compounds and gives results with high accuracy [25].

### 3.2. Molecular Docking Methods

Molecular docking study is applied to estimate the binding energy of ligands. Autodock Vina tools 1.5.6 [33] was used for the docking of the chosen phytochemicals, which are arbutin (P-1), carvacrol (P-2), coumarin (P-3), catechin (P-4), harmalol (P-5), harmaline (P-6), kaempferol (P-7), quercetin (P-8), vasicine (P-9), and vasicinone (P-10), as displayed in (Figure 1) [34]. The crystal structures of SARS-CoV-2 main protease (6LU7) [35] were obtained from the Protein Database (PDB) [36] and saved in a pdbqt file format before the docking process. 6LU7 was properly modeled by introducing polar hydrogens, Kollman charges, and fragment volume. In addition, a grid box (x = −10.729204, y = 12.417653, and z = 68.816122) was selected that surrounds the active site of protein. Using the auto-grid engine, the configuration file for the grid parameters (GPs) was created. The ligands and protein were considered flexible during the docking procedure. For further investigation, the optimal position with the highest docking level was selected [37].

### 3.3. Pharmacokinetic and Pharmacodynamics Investigations

Bioavailability measures the fraction of the administered dose that reaches the systematic circulation and is available to exert pharmacological effects, while metabolic stability assesses the susceptibility of a compound to metabolic degradation, which can affect its duration of action and overall efficacy [18]. The Swiss ADME (absorption, distribution, metabolism, and excretion) tool [38] was applied to determine the pharmacokinetic characteristics, such as the number of heavy atoms, ESOL Log S, ESOL, Solubility (mol/L), GI absorption and BBB permeability for the selected phytochemicals [39]. The Lipinski values of ligands were obtained by using PubChem database [40].

### 3.4. Molecular Dynamic Simulation Methods

As kaempferol from *Carica papaya* showed significant interactions with the target protein of COVID-19 in the experimental [41] and molecular docking study, so this compound was selected for further MD simulation. The stability of a protein–ligand complex was assessed using molecular dynamics (MD) simulations in a system under Na^+^ 33 (57.676 Mm) and ${Cl}^{-}$ 29 (50.685 Mm). Using Schrodinger’s Desmond molecular dynamics modeling program, the optimum compound, P-7-6LU7, was studied [42]. The OPLS_2005 force field was utilized to simulate the complex interactions between the protein and ligand. To ensure the accuracy of simulations, the complex was placed in a cubical water box (TIP3P water model), in three dimensions, with a 12 Å buffer space using Desmond’s system builder tool. Appropriate counter-ions were added to neutralize each system and were maintained by adding 0.15 M NaCl, ensuring a stable and consistent environment. With 10,000 steepest descent steps, the systems were minimized and gradually heated from 0 to 300 K, using NVT before each run. To ensure optimal results, the system was then thermally relaxed for 5 ns, using the Nose–Hoover Chain thermostat and Martyna–Tobias–Klein barostat methods for 5 ns of pressure relaxation. This was followed by keeping the constant particle number (N) at 4691 (total), heavy atoms at 2372, charge at −4, pressure (P) at 1.01325 bar, and ensemble conditions (NPT ensemble). The NPT ensemble was used to simulate 100 ns with a cutoff distance of 12 angstroms. To record the trajectories, 5000 frames were captured and saved at 4.8 picosecond intervals.

## 4. Conclusions

This study aimed to evaluate the potential of ten phytochemicals as inhibitors of SARS-CoV-2 Mpro spike protein targets. The results indicated that all ten phytochemicals interacted with the protein targets by docking in the cavity of the binding pocket, which contains common amino acid residues. The binding energy of all the phytochemicals was within an acceptable range, with kaempferol displaying the highest binding energy, i.e., −7.7 kcal/mol. The ProTox analysis revealed that these compounds were inert for most types of toxicity, except for coumarin (P-4), which showed hepatotoxicity and immunotoxicity; and quercetin (P-8), which was inactive against carcinogenicity. All phytochemicals obeyed Lipinski’s Rule of Five, suggesting that they could potentially be developed into drugs. Further MD simulations were performed to determine the stability of the interactions between the ligands and protein. The results suggested that kaempferol P-7 was the best-binding ligand, displaying stable interactions with the protein targets. Based on the pharmacokinetics and drug-like qualities, kaempferol (P-7) was identified as a potential candidate for developing novel medications. The phytochemical exhibited favorable properties, including high absorbability, solubility, and synthetic accessibility, indicating its potential as a drug candidate. Long-range interactions that are pivotal in complex biological processes are beyond the scope of molecular docking, which mainly focuses on short-range interactions within the binding sites. Further investigations might be enhanced by employing more comprehensive MD simulations, including extended simulation times and multiple replicates. In addition, it is imperative to conduct further experimental validations, including in vivo studies and binding affinity assays, to substantiate the in silico results and facilitate their translation into practical pharmaceutical applications.

## Figures and Tables

**Table 1:** Molecular formula (MF), the highest molecular orbital energies (E_HOMO_), the lowest molecular orbitals energies (E_LUMO_), HOMO-LUMO energy gap (H-L Egap), chemical hardness (*η*), electronic chemical potential (*µ*), electrophilicity index (ω), and nucleophilicity (N) of selected phytochemicals. All the values are given in eV.

Phytochemicals Codes	(MF)	E_HUMO_	E_LUMO_	H-L Egap	*η*	*µ*	*ω*	N
P-1	C_12_H_16_O_7_	−5.63	−0.02	5.60	−2.80	−2.83	2.02	−5.60
P-2	C_10_H_14_O	−5.74	−0.29	5.45	−2.73	−3.01	2.21	−5.45
P-3	C_15_H_14_O_6_	−6.52	−1.91	4.61	−2.31	−4.21	3.65	−4.61
P-4	C_9_H_6_O_2_	−5.50	−0.05	5.45	−2.73	−2.78	2.04	−5.45
P-5	C_12_H_12_N_2_O	−5.68	−0.79	4.89	−2.45	−3.24	2.65	−4.89
P-6	C_13_H_14_N_2_O	−5.10	−0.82	4.27	−2.14	−2.96	2.77	−4.27
P-7	C_15_H_10_O_6_	−5.54	−1.92	3.62	−1.81	−3.73	4.12	−3.62
P-8	C_15_H_10_O_7_	−5.43	−1.95	3.48	−1.74	−3.69	4.24	−3.48
P-9	C_11_H_12_N_2_O	−5.27	−0.50	4.77	−2.39	−2.89	2.42	−4.77
P-10	C_11_H_10_N_2_O_2_	−6.26	−1.11	5.15	−2.58	−3.68	2.86	−5.15

**Table 2:** SAR analysis, including surface area (Grid) (Å^2^), volume (Å^3^), hydration energy (kcal/mole), Log P, refractivity (Å^3^), and polarizability (Å^3^) of considered phytochemicals (P-1 to P-10).

Functions	P-1	P-2	P-3	P-4	P-5	P-6	P-7	P-8	P-9	P-10
Surface area (Grid) (Å^2^)	4.73 × 10^2^	7.61 × 10^2^	1.10 × 10^3^	7.73 × 10^2^	8.78 × 10^2^	9.36 × 10^2^	2.73 × 10^1^	1.00 × 10^3^	4.48 × 10^1^	9.22 × 10^2^
Volume (Å^3^)	7.83 × 10^2^	1.45 × 10^3^	2.14 × 10^3^	1.28 × 10^3^	1.77 × 10^3^	1.84 × 10^3^	2.02 × 10^3^	2.02 × 10^3^	2.15 × 10^1^	1.75 × 10^3^
Hydration energy (kcal/mole)	−2.23 × 10^1^	−3.26 × 10^5^	−3.71 × 10^6^	−3.71 × 10^6^	−3.34 × 10^6^	−1.04 × 10^6^	−6.37 × 10^0^	−1.68 × 10^5^	1.338 × 10^1^	−7.94 × 10^4^
Log P	−9.55 × 10^−1^	−2.95 × 10^1^	−4.80 × 10^1^	−2.64 × 10^1^	−3.83 × 10^1^	−4.20 × 10^1^	−4.54 × 10^1^	−4.59 × 10^1^	−6.50 × 10^−1^	−3.51 × 10^1^
Refractivity (Å^3^)	6.34 × 10^1^	8.74 × 10^1^	1.36 × 10^2^	7.23 × 10^1^	1.09 × 10^2^	1.19 × 10^2^	1.29 × 10^2^	1.33 × 10^2^	1.60 × 10^0^	1.03 × 10^2^
Polarizability (Å^3^)	2.57 × 10^1^	3.83 × 10^1^	5.84 × 10^1^	3.10 × 10^1^	4.63 × 10^1^	5.04 × 10^1^	5.53 × 10^1^	5.68 × 10^1^	7.70 × 10^−1^	4.35 × 10^1^

**Table 3:** Binding affinity analysis of phytochemicals (P-1 to P-10) with 6LU7 across the three more stable orientations.

Complex	Binding Affinity (kcal/mol)		
	1st Most Stable Orientation	2nd Most Stable Orientation	3rd Most Stable Orientation
P-1@6LU7	−6.8	−6.6	−6.5
P-2@6LU7	−4.7	−4.6	−4.6
P-3@6LU7	−7.2	−7.1	−7.1
P-4@6LU7	−5.0	−5.0	−5.0
P-5@6LU7	−6.3	−6.0	−5.9
P-6@6LU7	−6.3	−6.0	−5.7
P-7@6LU7	−7.7	−7.3	−7.1
P-8@6LU7	−7.5	−7.4	−7.4
P-9@6LU7	−5.7	−5.3	−5.3
P-10@6LU7	−5.9	−5.8	−5.7

**Table 4:** In silico analysis of the pharmacokinetics and pharmacodynamics properties of phytochemicals (P-1–P-10), including molecular weight (MW), hydrogen bond donor (HBD), Lipinski Rule violation (L), hydrogen bond acceptor (HBA), rotatable bond (RB), synthetic accessibility (SA_score)_, (estimated solubility) ESOL Log S, Estimated solubility (ESOL), gastrointestinal (GI) absorption, and blood–brain barrier (BBB) permeability.

Phytochemical’s Codes	MW	L	HBD	HBA	RB	SA_score_	ESOL Log S	ESOL Solubility (mol/L)	GI	BBB
P-1	272.25	0	5	7	3	4.18	−0.71	1.94 × 10^−1^	High	No
P-2	150.22	0	1	1	1	1	−3.31	4.92 × 10^−4^	High	Yes
P-3	290.27	0	0	2	0	2.74	−2.29	5.08 × 10^−3^	High	Yes
P-4	146.14	0	5	6	1	3.50	−2.22	5.08 × 10^−3^	High	No
P-5	200.24	0	2	2	0	2.51	−2.63	2.36 × 10^−3^	High	Yes
P-6	214.26	0	1	2	1	2.58	−2.82	1.50 × 10^−3^	High	Yes
P-7	286.24	0	6	4	1	3.14	−3.31	4.90 × 10^−4^	High	No
P-8	302.24	0	5	7	1	3.23	−3.16	6.98 × 10^−4^	High	No
P-9	188.23	0	1	2	0	3.36	−1.60	2.50 × 10^−2^	High	No
P-10	202.21	0	1	3	0	2.75	−1.91	1.22 × 10^−2^	High	No

## Data Availability

Data will be made available to the corresponding author upon request.

## Supplementary Materials

The following supporting information can be downloaded at: https://www.mdpi.com/article/10.3390/molecules29051144/s1, Figure S1: Two-dimensional (a), three-dimensional (b) and residue interactions analysis (c) based structures of P-1 to P-10 complexes except for P7-6LUcomplex.
