# Supplementary material for: SAR, Molecular Docking and Molecular Dynamic Simulation of Natural Inhibitors against SARS-CoV-2 Mpro Spike Protein
Source: Molecules. 2024 Mar 4;29(5):1144. doi: 10.3390/molecules29051144 (PMC10934104; doi:10.3390/molecules29051144)
Supplement: Supplementary file 1 [file molecules-29-01144-s001.zip › molecules-2817424-supplementary.pdf]

Supporting information

for

SAR, Molecular Docking and Molecular Dynamic Simulation of Natural inhibitors against SARS-CoV-19 Mpro spike protein

Arbutin (P-1)

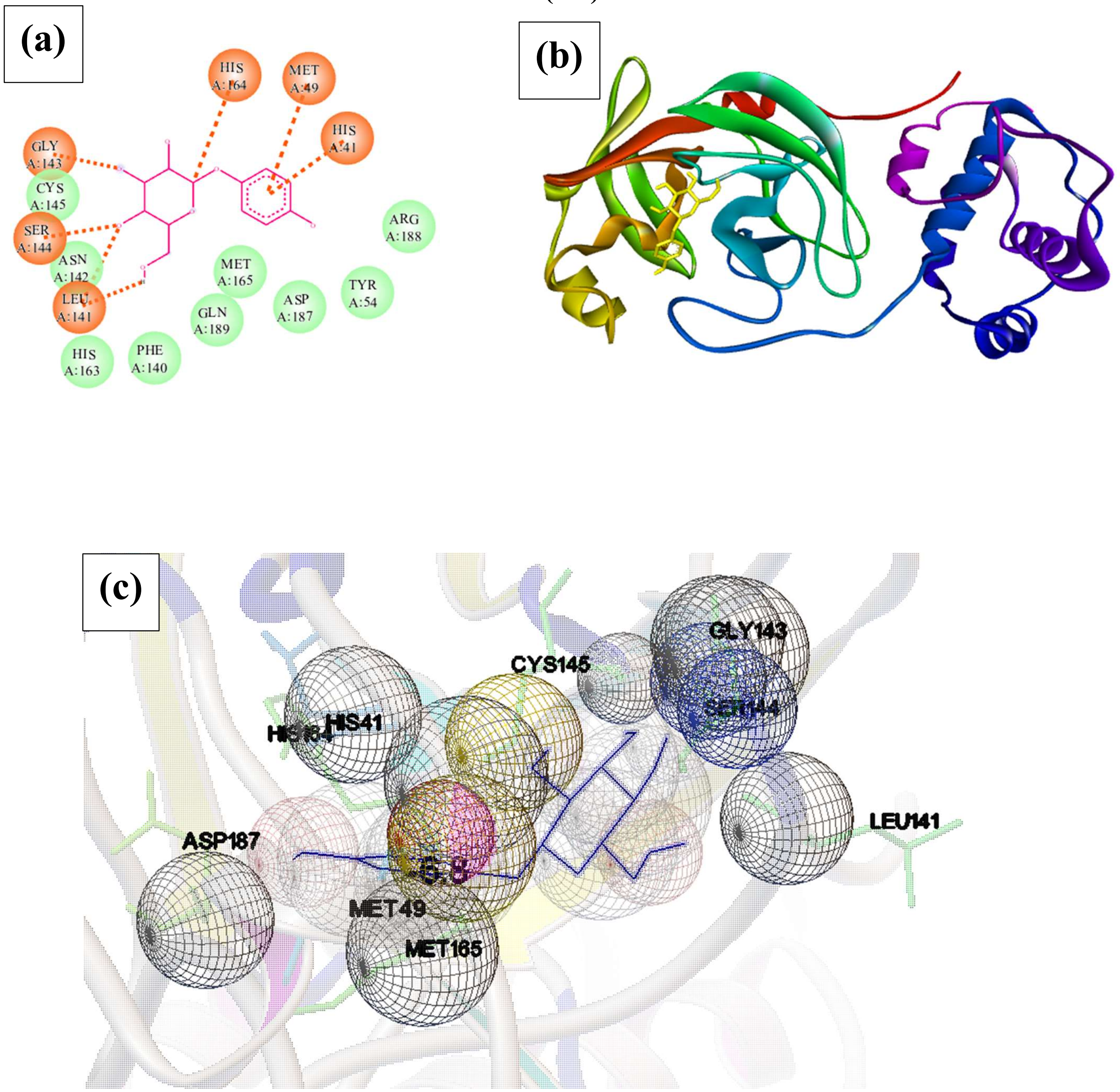

Carvacrol P-2

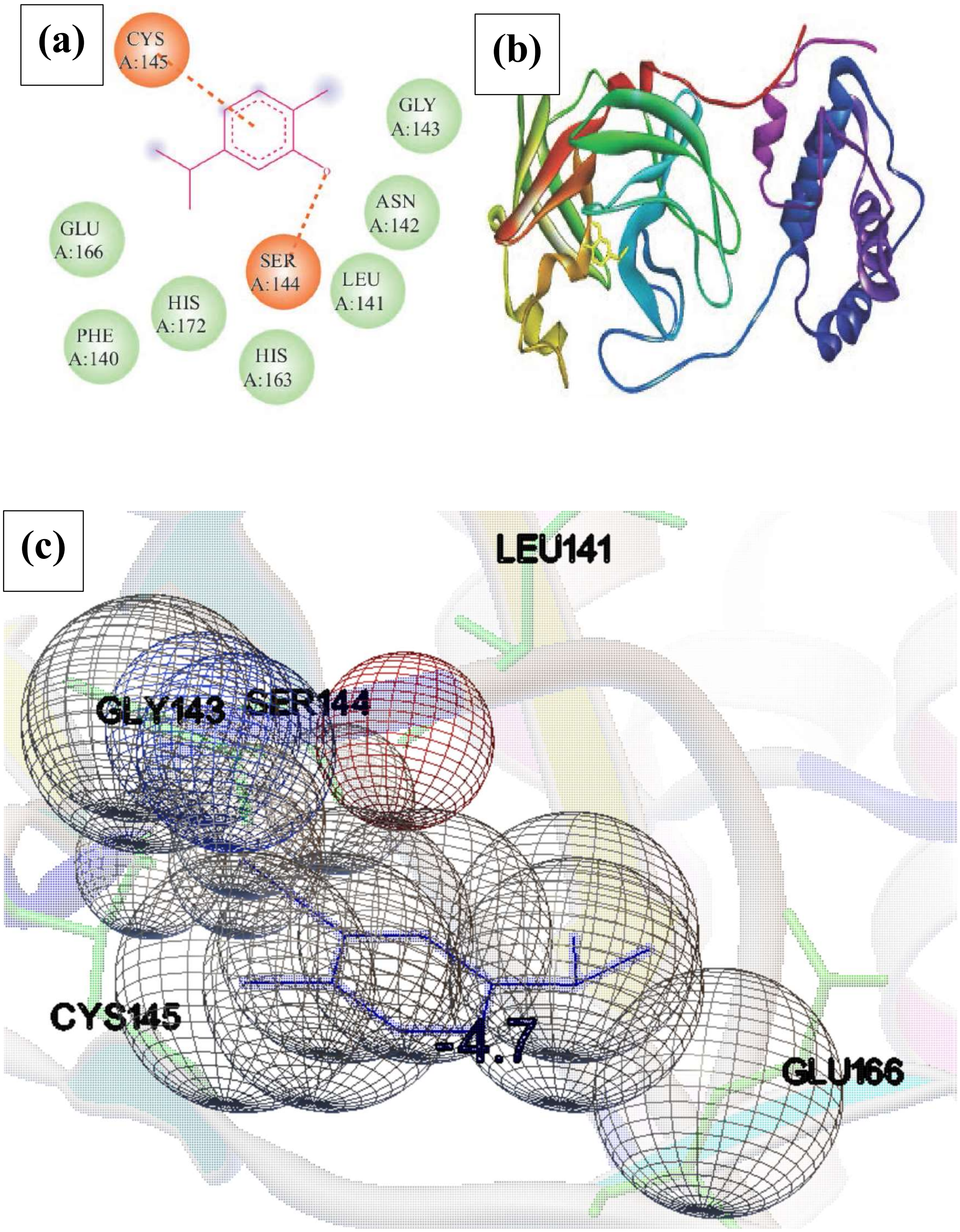

Coumarin P-3

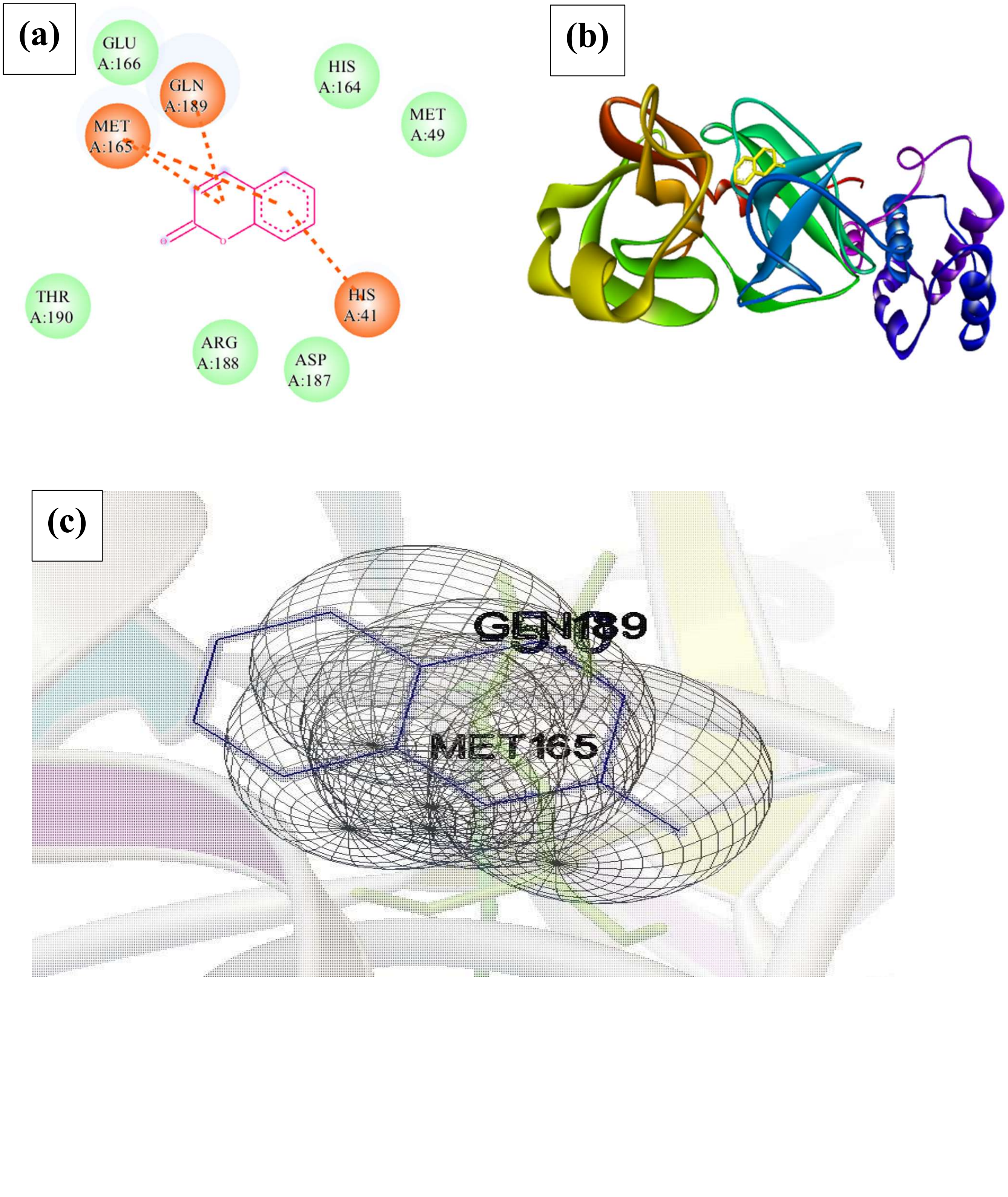
(a)
GLU A:166
GLN A:189
HIS A:164
MET A:49
MET A:165
THR A:190
HIS A:41
ARG A:188
ASP A:187
(b)
(c)
MET165

Catechin P-4

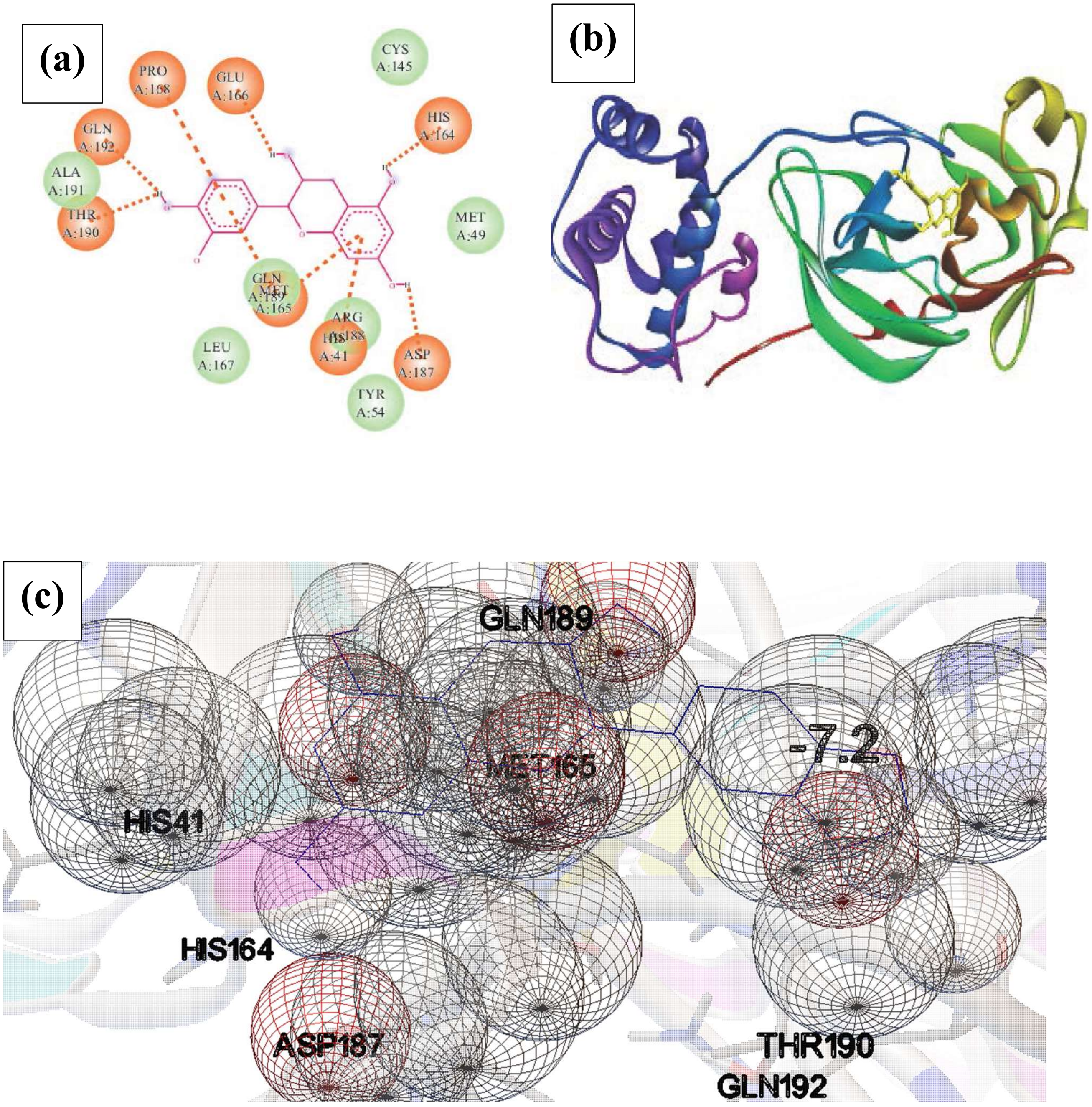
(a)
PRO A:168
GLU A:166
CYS A:145
HIS A:164
GLN A:192
ALA A:191
THR A:190
MET A:49
GLN A:189
MET A:165
ARG A:188
HIS A:41
LEU A:167
ASP A:187
TYR A:54
(b)
(c)
GLN189
MET165
-7.2
HIS41
HIS164
ASP187
THR190
GLN192

Harmalol P-5

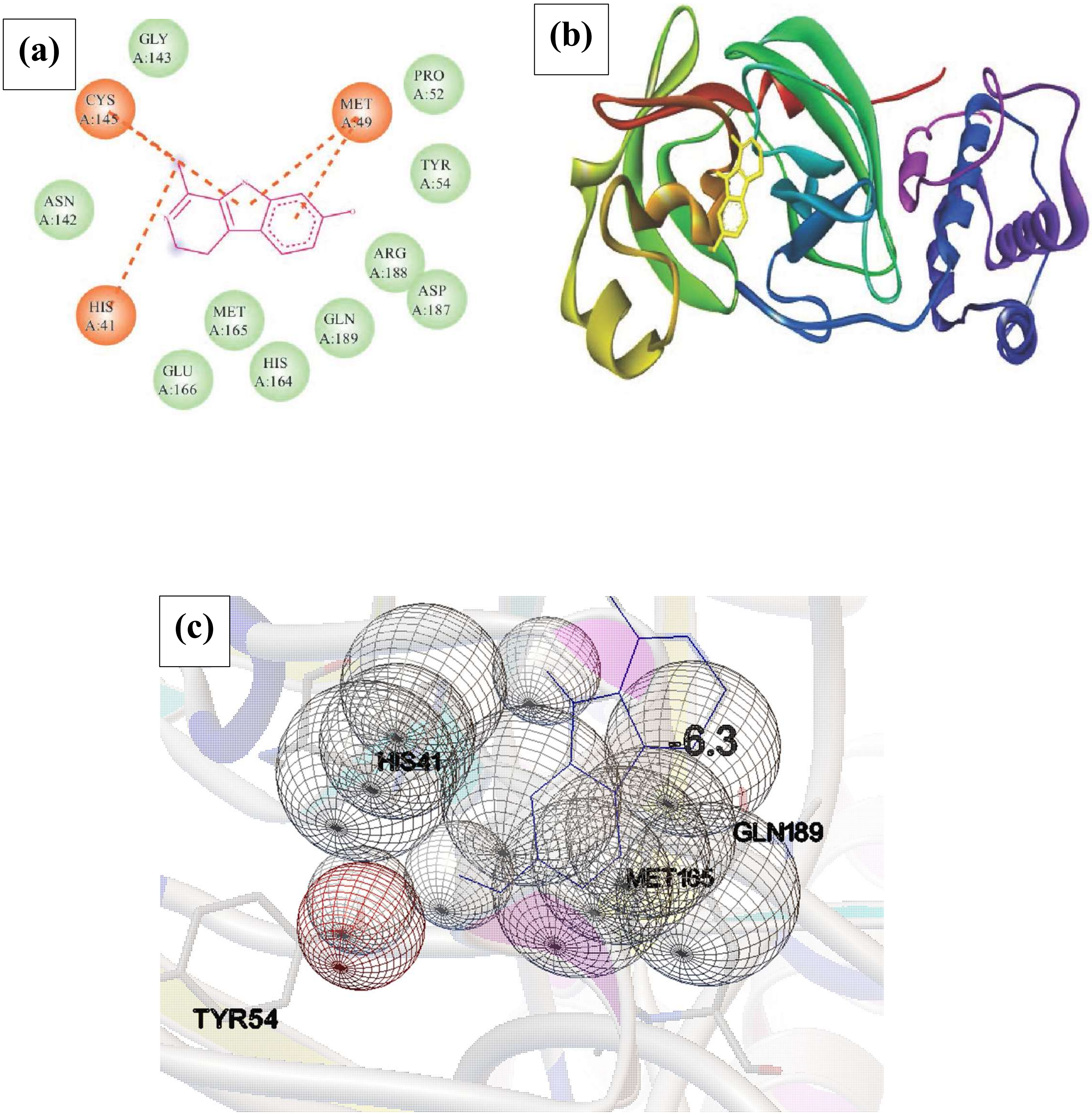

(a)
GLY A:143
CYS A:145
MET A:49
PRO A:52
TYR A:54
ASN A:142
ARG A:188
ASP A:187
HIS A:41
MET A:165
GLN A:189
GLU A:166
HIS A:164
(b)
(c)
HIS41
-6.3
GLN189
MET165
TYR54

Harmaline P-6

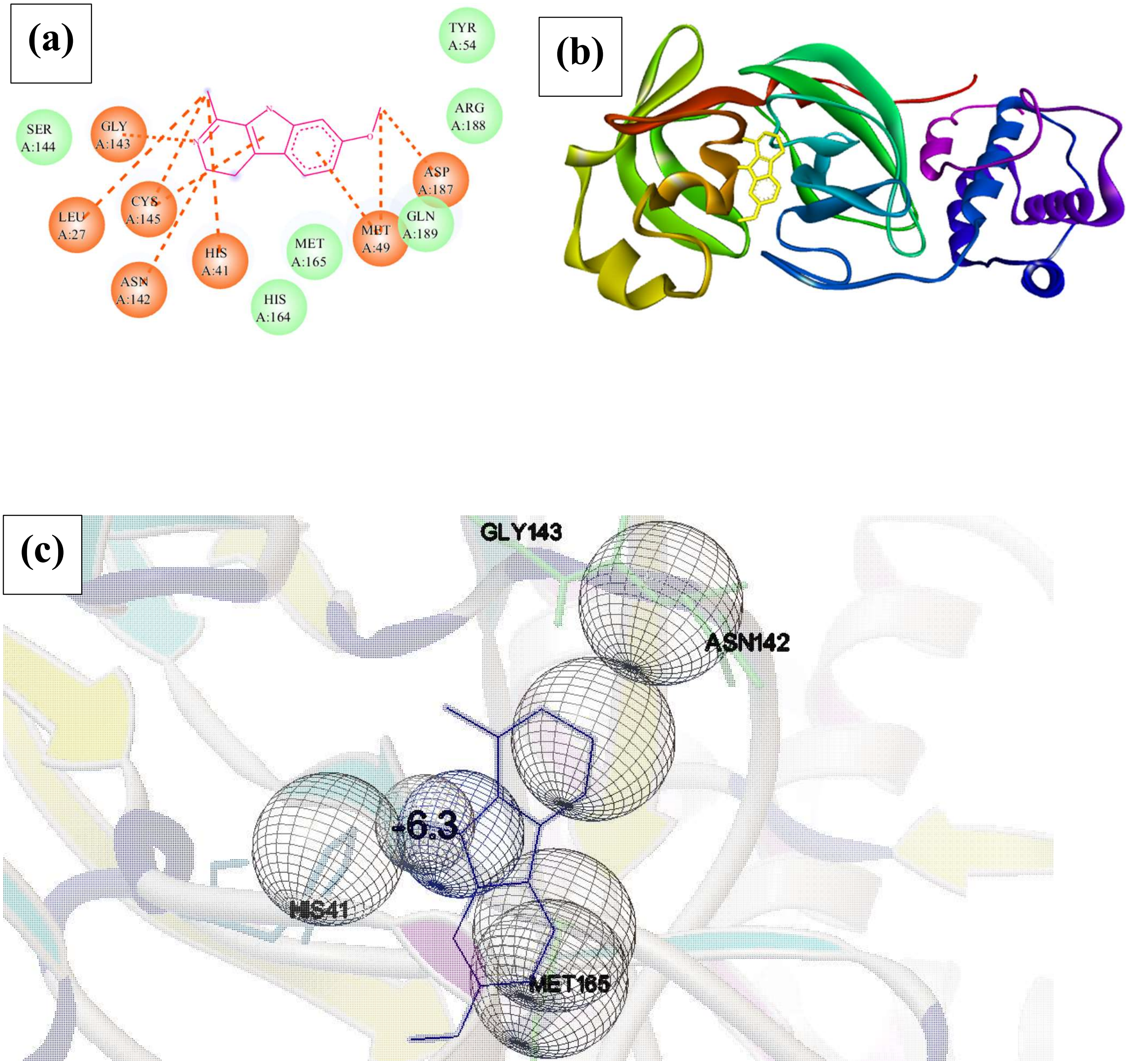

Quercetin P-8

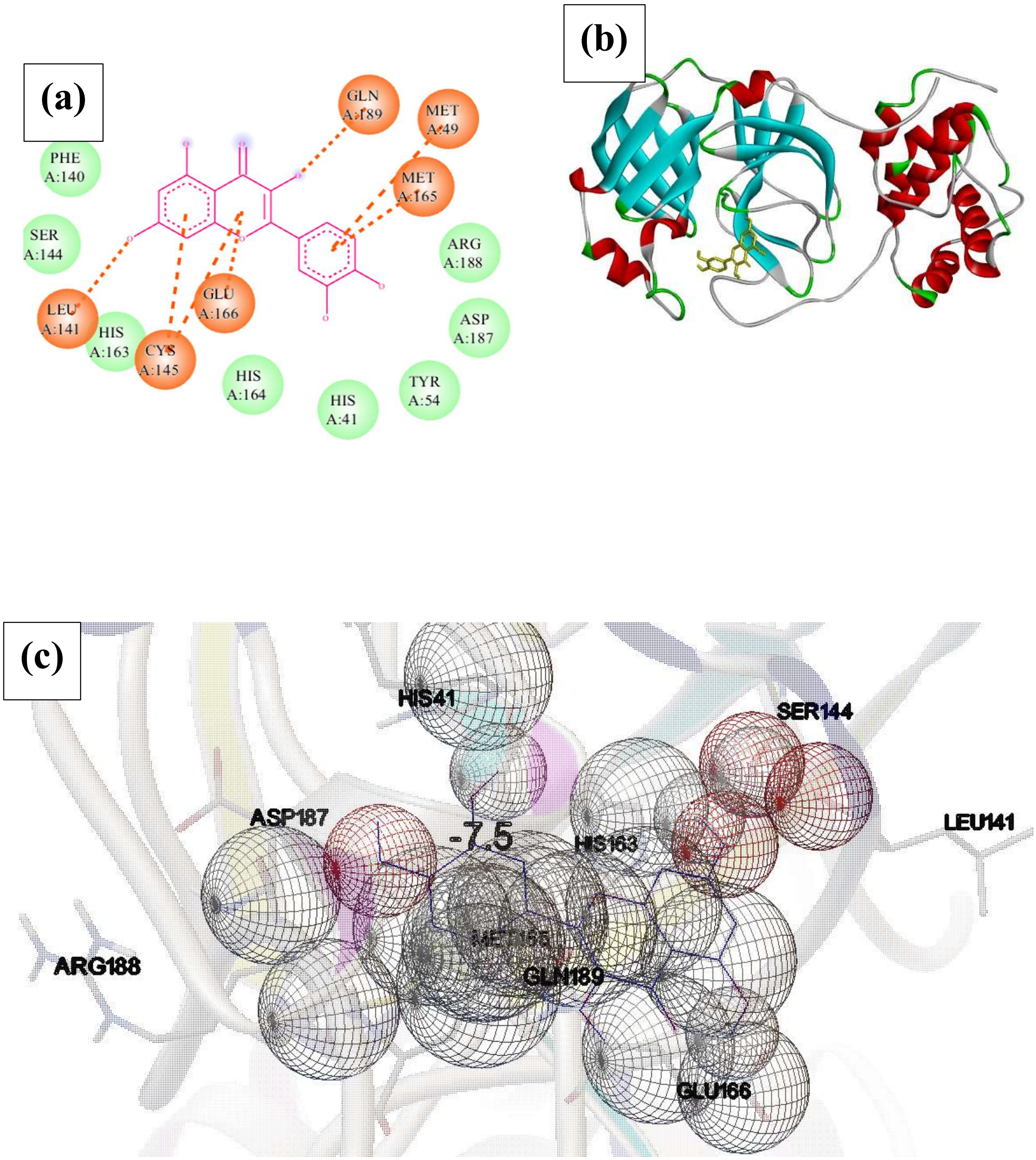

Vasicine P-9

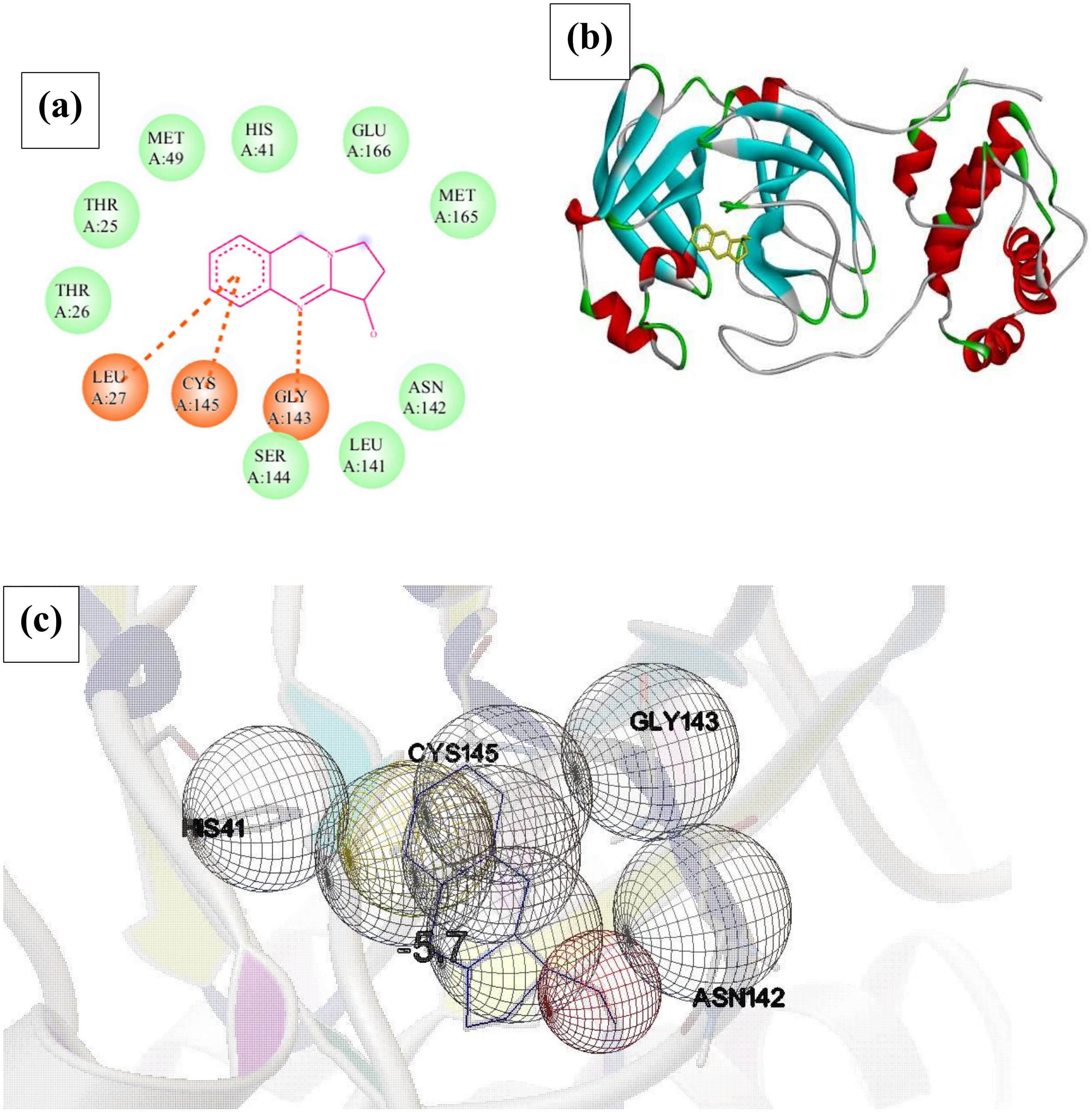
(a)
MET A:49
HIS A:41
GLU A:166
MET A:165
THR A:25
THR A:26
LEU A:27
CYS A:145
GLY A:143
ASN A:142
SER A:144
LEU A:141
(b)
(c)
CYS145
GLY143
HIS41
-5,7
ASN142

Vasicinone P-10

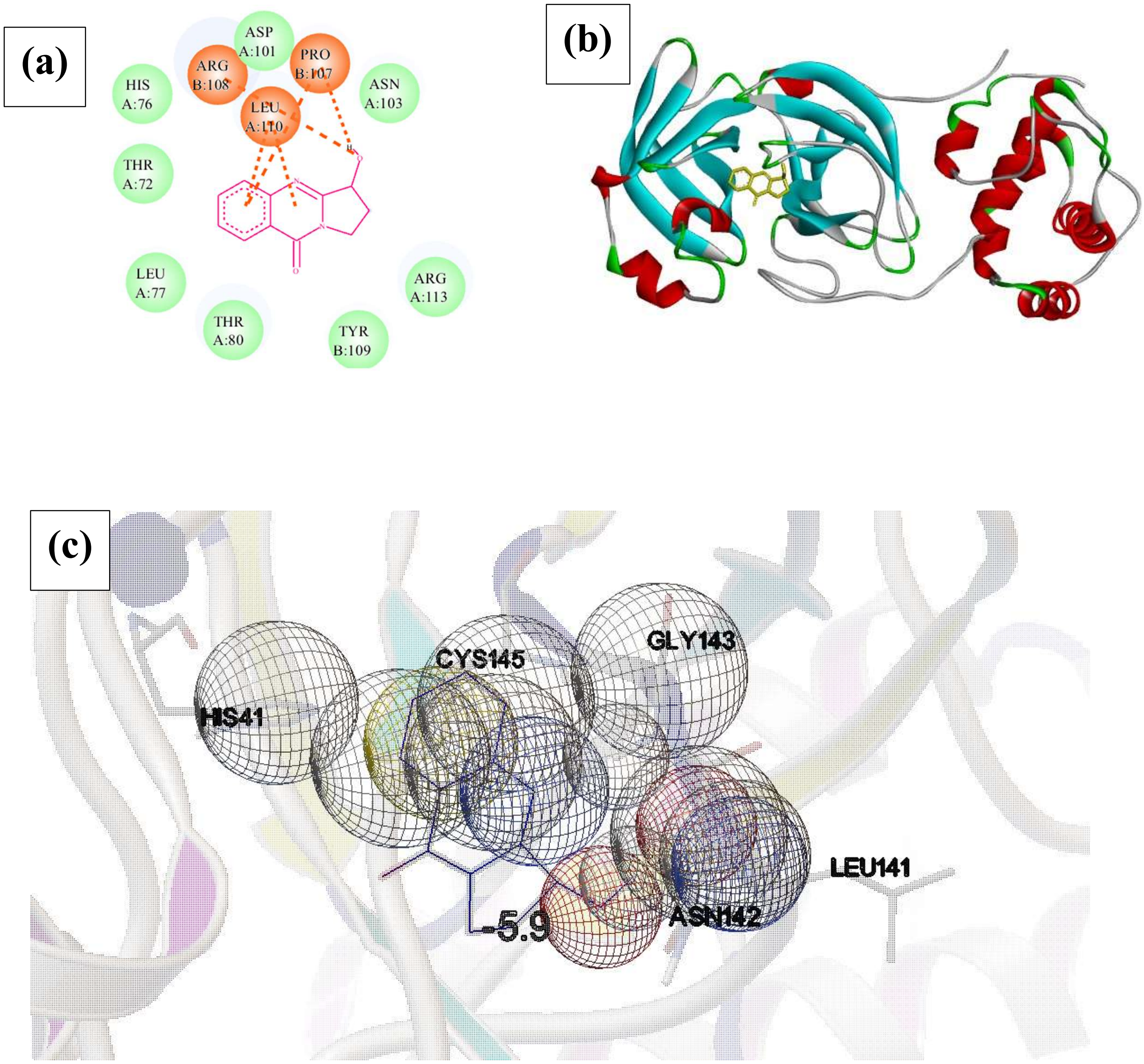

Fig. SI. 1: Two-dimensional (a), three-dimensional (b) and residue interactions analysis (c) based structures of **P-1** to **P-10** complexes except for **P7-6LU7** complex.
